# Comparison between volume-controlled ventilation and pressure-controlled volume-guaranteed ventilation in postoperative lung atelectasis using lung ultrasound following upper abdominal laparotomies: a prospective randomized study

**DOI:** 10.1186/s42077-020-00076-9

**Published:** 2020-07-14

**Authors:** Bahaa El-din Ewees Hassan, Ahmed Nagah El-Shaer, Marwa Ahmed Khairy Elbeialy, Shimaa Ahmed Mohamed Ismail

**Affiliations:** 1 Department of Anesthesiology, Intensive Care and Pain Management, Faculty of Medicine, Ain-Shams University, Abbassia, Cairo, 11591 Egypt; 2 Faculty of Medicine, Ain-Shams University, Cairo, Egypt

**Keywords:** Ventilation, Atelectasis, Ultrasound, Laparotomy, Abdominal

## Abstract

**Background:**

Atelectasis is a common side effect of general anesthesia. Prevention of lung atelectasis, carbon dioxide retention, and chest infection would improve the quality of medical care and decrease hospital stay and costs. The aim of this study was to compare the effects of volume-controlled ventilation (VCV) and pressure-controlled volume-guaranteed ventilation (PCVG) on postoperative lung atelectasis using lung ultrasound (LUS) following upper abdominal laparotomies.

**Results:**

Sixty patients (male and female) scheduled for upper abdominal laparotomies. They were randomly allocated into two equal groups: Group A (*n* = 30): received intraoperative volume-controlled ventilation (VCV) mode and group (*n* = 30): received intraoperative pressure-controlled ventilation volume-guaranteed (PCV-VG) mode. Arterial blood samples were obtained immediately after extubation, and 30, 120, 240, and 360 min postextubation. Lung ultrasound was done intraoperatively at 30 min from induction, immediate, and 120 and after 360 min postoperatively. There was difference between two groups favoring PCV-VG group but that difference failed to be statically significant regarding arterial partial pressure of oxygen (PaO_2_) and arterial carbon dioxide tension (PaCo_2_) between the two groups in preoperative, immediate postoperative, and 120, 240, and 360 min postoperative. Arterial oxygen saturation (SaO_2_) was significantly lower among patients in the VCV group immediate postextubation compared with patients in group PCV-VG (*p* value = 0.009*). Although signs of atelectasis were low in group B, 36.7% of the patients showed normal lung ultrasound, 63.3% showed various abnormalities, 46.7% showed the presence of lung pulse (vertical rhythmic movement synchronous with cardiac pulsation through motionless lung), and 46.7% showed B lines (vertical lines indicate abnormal lung aeration), while 30% of the patients showed the absence of A-lines (indicates the absence of lung sliding and abnormal lung aeration). Also, some patients demonstrated more than one sign. However, there was no a significant difference between the two groups both showed atelectasis immediate, 2 h and 6 h postoperatively.

**Conclusion:**

PCV-VG offered no significant advantage over VCV regarding the occurrence of the postoperative atelectasis. However, we prefer to use PCV-VG as postoperative hypoxia and atelectasis was much less in that mode. Further, large-scale studies are required to confirm these findings and to establish a definite conclusion.

## Background

General anesthesia causes depression in both respiratory centers and respiratory muscles. Hence, patients under general anesthesia require adequate ventilatory support to maintain arterial oxygenation and eliminate carbon dioxide (Hedenstierna & Edmark, 2010).

Atelectasis formation during general anesthesia is the main cause of the increase in intrapulmonary shunt (Magnusson & Spahn, 2003) immediately after the induction of general anesthesia, leading to a reduction in both ventilation to perfusion ratio and pulmonary compliance even in non-obese patients (Duggan & Kavanagh, 2015).

Volume-controlled ventilation (VCV) is the most commonly used modes. In this mode, a preset tidal volume (TV) with a constant flow during the preset inspiratory time (Ti) at the preset respiratory rate is delivered with each breath. The disadvantages of VCV include a higher airway pressure at the end of inspiration than during pressure-controlled ventilation (PCV) (Santanilla et al., 2008).

In PCV-VG, the TV and the respiratory rate are predetermined and the ventilator delivers the TV using a decelerating flow but a constant pressure. The ventilator adjusts the inspiratory pressure needed to deliver the TV breath-by-breath so that the lowest pressure is used. PCV-VG begins by first delivering a volume breath at the set TV. The patient’s compliance is determined from this volume breath, and the inspiratory pressure level is then established for the next breath. Hence, PCV-VG combines the benefits of decelerating the flow of PCV with the safety of a volume guarantee at a lowest possible titrated inspiratory pressure (Keszler, 2006).

Recently, a specific ultrasonographic detection of the cardiac impulse definition termed the lung pulse is being used as a highly sensitive early indicator of the presence of atelectasis. Lung pulses are tenuous, rhythmic, vertical movement of visceral upon parietal pleurae synchronous with cardiac pulsations, and are caused by the transmission of cardiac oscillations through the motionless lung and can be objectified on M-mode (Copetti et al., 2008).

The sensitivity and specificity of LUS to detect atelectasis are 93% and 100%, respectively. Also, the absence of lung sliding with lung pulse and standstill cupola (absence of lung expansion) is the early signs of atelectasis. With progressive absorption of air, there is a loss of volume leading to a hypoechoic pattern known as static air Broncho gram (late sign) (Lichtenstein et al., 2009).

Lung ultrasonography in the perioperative period is feasible, allows tracking of perioperative atelectasis, and facilitates the diagnosis of respiratory complications. The evolution of aeration loss correlates moderately with changes in oxygenation (Monastesse et al., 2017).

We hypothesized that the PCV-VG mode of ventilation with its decelerating flow pattern would result in a significant improvement in PaO_2_ and/or CO_2_ removal. Also, it would decrease the occurrence of postoperative basal lung atelectasis.

This study aims to compare the effects of VCV and PCV-VG on postoperative lung atelectasis using chest ultrasound following upper abdominal laparotomies.

## Methods

After approval of the Research Ethical Committee of the Faculty of Medicine, Ain Shams University, and obtaining informed consent, this prospective randomized comparative study was conducted in Ain Shams University Hospitals. Sixty patients (male and female) scheduled for upper abdominal laparotomies lasting 3 to 6 h were included in this study. They were randomly allocated into two equal groups using computer-generated randomized table and sealed opaque envelopes. Group A (*n*=30): received intraoperative volume controlled ventilation (VCV) mode and group B (*n*=30): received intraoperative pressure controlled ventilation – volume guaranteed (PCV- VG) mode.

The study included patients of either sex with normal preoperative pulmonary function, a satisfactory pulmonary function tests (defined as > 70% of predicted values for pulmonary function test) preoperative PaCO_2_ and PaO_2_, who were scheduled for elective upper abdominal laparotomies such as open cholecystectomy and splenectomy.

Patients who refused to participate in the study, those who had pulmonary, severe hepatic, cardiac or renal diseases, or had body mass index (BMI) more than 25, a position rather than supine or a duration less than 3 h were excluded from the study. If any of the patients experienced hemodynamic instability such as massive hemorrhage, he/she was omitted from study.

### Study procedures

#### Preoperative evaluation

Patients underwent a thorough preoperative assessment including a detailed history, physical examination, laboratory investigations, and chest X-ray evaluation. All patients included in the study were fasting for at least 8 h before the induction of anesthesia. Baseline arterial blood gases (ABG) for PaCO_2_, PaO_2_, and SaO_2_ were obtained, and baseline lung ultrasound was performed before the operation.

Patients received 0.02 mg/kg intravenous (IV) midazolam as premedication. Prophylaxis for postoperative nausea and vomiting included intravenous ondansetron (4 mg). In the operating room (OR), standard monitoring in the form of a five-lead electrocardiogram, noninvasive blood pressure, pulse oximetry, and capnography were established.

Induction of anesthesia was done by preoxygenation followed by 1–2 mg/kg IV propofol, 1–2 μg/kg IV fentanyl, 0.5 mg/kg IV atracurium to facilitate tracheal intubation. Maintenance of anesthesia was achieved by 0.5–1 MAC of isoflurane in a 50% oxygen/air mixture and 0.15 mg/kg IV atracurium boluses guided by a nerve stimulator, and the patients received fluid requirements. Fluid intake was in the form of crystalloid.

All patients were mechanically ventilated using Datex-Omeda ventilator, VCV, or PCV-VG mode according to the allocated group throughout the surgery with a TV of 6–8 ml/kg of ideal body weight, and the respiratory rate was adjusted to maintain normocapnia (ETCO_2_ 32-35 mmHg) and well oxygenation I to E ratio set at 1:2 and P max at 40 cm H_2_O. A positive-end expiratory pressure (PEEP) of 5 cm H_2_O was added in both modes throughout the surgery. FiO_2_ was 0.6 throughout the whole operation. In the VCV group, the ventilator settings were continued throughout the study. In the PCV-VG group, PCV-VG mode was chosen to achieve VT 6–8 mL/kg, with RR adjusted to maintain ETCO_2_ 32–35 mmHg. Intraoperative LUS was performed 30 min after induction. Recovery was carried out after closure of the surgical wound by turning off isoflurane vaporizer and increasing FiO_2_ to 1.0. When respiratory attempts started, neostigmine (0.02–0.04 mg/kg) and atropine (0.02 mg/kg) were given to reverse residual neuromuscular block. This was followed by extubation when awake and patient transferal to Post-Anesthesia Care Unit (PACU). The patients were put in the semi-setting position at the PACU with supplementation of oxygen face mask, and then, the patient was discharged to the ward after fulfilling the recovery criteria. Postoperative analgesia was provided to keep patients with a visual analog scale (VAS) less than 3 in the form of paracetamol (10–15 mg/kg/6 h) and morphine sulfate (0.05–0.1 mg/kg/Iv) every 6 h. If VAS > 4, top-up doses of morphine were given with max dose 5 mg/6 h for all patients. Arterial blood samples were withdrawn to measure PaCO_2_, PaO_2_, and SaO_2_ immediately after extubation. ABG analysis was also obtained at 120, 240, and 360 min postextubation.

Lung ultrasound was performed using a curved probe was performed to each group of patients postoperatively, immediately postextubation, and then 120 and 360 min later in a supine position placing the probe on the lateral and inferior chest wall and longitudinally.

According to the systematic protocol for LUS examination (Bouhemad et al., 2015), the LUS of a normal lung shows a lung sliding (caused by the respiratory movement of the visceral pleura relative to the fixed parietal pleura) and A-lines (repetitive horizontal reverberation artifacts generated by air within the lungs separated by regular intervals, the distances of which being equal to that between the skin and the pleural line). Detection of B lines (vertical lines indicate abnormal lung aeration), Lung pulse (vertical rhythmic movement synchronous with cardiac pulsation through motionless lung), absences of lung sliding and A lines suggest presence of lung Atelectasis. Early detection of postoperative atelectasis will minimize postoperative pulmonary complication (PPC) events.

The primary outcome was detecting postoperative atelectasis following both modes using LUS; secondary outcomes were detecting changes in PaCO_2_, and SpO_2_ postoperative between two modes.

##### Sample size

Using the PASS program, settling alpha error at 0.05 and power at 80% results from previous study (El-Rawas et al. 2015) (El-Rawas et al., 2015) showed no significant difference between mean SpO_2_ in two studied groups (98.7 ± 0.85 vs 98.6 ± 0.86). Considering a non-inferiority study with a margin of non-inferiority, we estimated that 30 patients in each group were needed to demonstrate a statistically significant difference with a total number of 60 patients. The effect size equals 0.11 (small effect size).

### Statistical analysis

Data was analyzed using SPSS win statistical package version 20 (Statistical Packages for Social Science, Chicago, IL, USA). Numerical data were expressed as mean and standard deviation or median and range as appropriate. Qualitative data were expressed as frequency and percentage. For quantitative data, the comparison between two groups was done using either student *t* test or Mann-Whitney test (non-parametric *t* test) as appropriate. Values of pre- and postassessments were analyzed by paired *t* test or Wilcoxon signed-rank test as appropriate. Chi-square test used to examine the relation between qualitative variables. The confidence interval was set to 95%, and the margin of error accepted was set to 5%. So, the *p* value was considered significant if < 0.05.

## Results

**Fig. 1:**
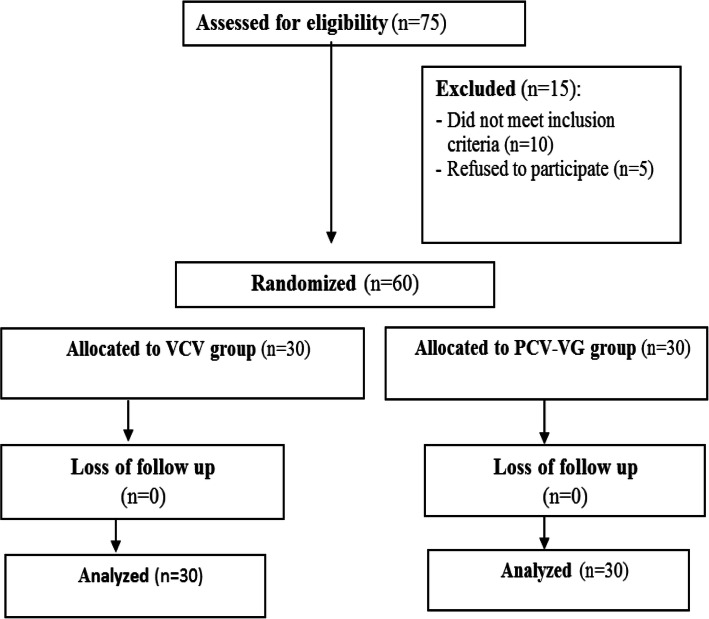
Patient flow chart

Seventy-five patients were screened for this study, 10 patients did not meet inclusion criteria and 5 patients refused to participate in our study. The remaining 60 patients were randomly allocated to the groups of the study (Fig. 1).

### Patient data

There was no significant difference between the three groups regarding age, gender, and duration of surgery (Table 1).

**Table 1:** Comparison between group A: VCV and group B: PCV-VG according to the demographic data

Demographic data	Group A: VCV ***(n = 30)***	Group B: PCV-VG ***(n = 30)***	***t***/***x***^**2**^#	***p*** value
**Age (years)**				
Mean ± SD	31.20 ± 7.18	30.26 ± 6.96	1.735	0.116
**Gender**				
Male	9 (30.0%)	7 (23.3%)	1.314#	0.256
Female	21 (70.0%)	23 (76.7%)		
**Duration of surgery (min)**				
Mean ± SD	140.40 ± 25.69	136.19 ± 24.92	0.229	0.620

Data presented as mean ± SD

*t* independent sample *t* test, *#x*^*2*^ chi-square test, *P* value > 0.05 NS

*P value < 0.05 S

**P value < 0.001 HS

### Type of surgery

There was no statistically significant difference between groups regarding the type of surgery (Table 2).

**Table 2:** Comparison between group A: VCV and group B: PCV-VG according to the type of surgery

Type of surgery	Group A: VCV ***(n = 30)***	Group B: PCV-VG ***(n = 30)***	***x***^**2**^	***p*** value
Panhysterectomy	12 (40.0%)	13 (43.3%)	0.052	0.862
Ovariectomy	8 (26.7%)	9 (30.0%)	0.052	0.862
Right hemicolectomy	2 (6.7%)	2 (6.7%)	0.000	1.000
Partial gastrectomy	2 (6.7%)	1 (3.3%)	0.052	0.862
Open cholecystectomy	4 (13.3%)	3 (10.0%)	0.052	0.862
Spleenectomy	2 (6.7%)	2 (6.7%)	0.000	1.000

Data are presented as the number of cases and the percentage of each to the total number of cases in each group

*x*^*2*^ chi-square test; *P value > 0.05 NS*

### Preoperative spirometer

There was no statistically significant difference between groups regarding the preoperative spirometer (Table 3).

**Table 3:** Comparison between group A: VCV and group B: PCV-VG according to the preoperative spirometer

Lung volumes	Group A: VCV ***(n = 30)***	Group B: PCV-VG ***(n = 30)***	***t***/***x***^**2**^#	***p*** value
**FEV1**	**2.38 ± 0.7**	**2.48 ± 0.5**	**0.637**	**0.526**
**FVC**	**3.36 ± 0.8**	**3.5 ± 0.8**	**0.678**	**0.5**
**FEV1/FVC (%)**	**71.4 ± 10.3**	**74 ± 11**	**0.945**	**0.348**
**TLC**	**5.4 ± 1.0**	**5.3 ± 1.2**	**-0.351**	**0.727**

Data presented as mean ± SD

*FEV1* forced expiratory volume in the first second, *FVC* forced vital capacity, *t* independent sample *t* test; *#x*^*2*^ chi-square test

*p* value *>* 0.05 NS; *p value < 0.05 S; **p value < 0.001 HS

### PaO_2_

There was no significant difference in PaO_2_ between the two groups in preoperative; immediate postoperative; and 30, 120, 240, and 360 min postoperatively (Table 4).

**Table 4:** Comparison between group A: VCV and group B: PCV-VG according to PaO_2_

PaO_**2**_	Group A: VCV ***(n = 30)***	Group B: PCV-VG ***(n = 30)***	***t*** test	***p*** value
Preoperative	85.50 ± 10.09	85.83 ± 8.31	0.138	0.890
After induction	83.79 ± 9.89	85.66 ± 8.14	0.800	0.427
Immediate postoperative	81.54 ± 8.06	85.57 ± 14.25	1.348	0.182
Postoperative at 30 min	81.9 ± 7.03	86.02 ± 13.40	1.491	0.141
Postoperative at 120 min	83.59 ± 8.40	83.03 ± 14.06	0.187	0.852
Postoperative at 240 min	84.52 ± 7.73	85.5 ± 12.07	0.374	0.709
Postoperative at 360 min	85.72 ± 9.01	86.85 ± 13.76	0.376	0.708

Data presented as mean ± SD and numbers as appropriate; *p* value < 0.05 was considered statistically non-significant

### SaO_2_

**Fig. 2:**
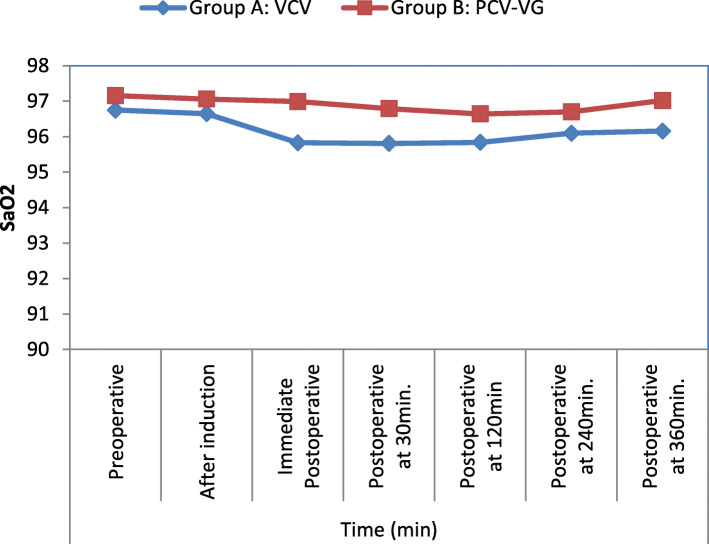
Comparison between group A: VCV and group B: PCV-VG according to SaO_2_

There was no significant difference in SaO_2_ between the two groups in pre-operative, immediate post-operative, and at 30,120, 240, and 360 min post-operatively. SaO_2_ was significantly lower among patients in the VCV group immediate postoperatively compared with patients in group PCV-VG (*p* value = 0.009*) and after 30 min (*p* value = 0.0107) (Table 5) (Fig. 2).

**Table 5:** Comparison between group A: VCV and group B: PCV-VG according to SaO_2_

SaO_**2**_	Group A: VCV (***n*** = 30)	Group B: PCV-VG (***n*** = 30)	***t*** test	***p*** value
Preoperative	96.75 ± 1.45	97.16 ± 1.37	1.126	0.264
After induction	96.65 ± 1.47	97.06 ± 1.30	1.144	0.257
Immediate postoperative	95.83 ± 1.85	96.99 ± 1.45	2.703	0.009*
Postoperative at 30 min	95.81 ± 1.55	96.79 ± 1.32	2.637	0.0107*
Postoperative at 120 min	95.84 ± 2.24	96.64 ± 2.23	1.386	0.171
Postoperative at 240 min	96.10 ± 2.70	96.70 ± 2.48	0.896	0.373
Postoperative at 360 min	96.16 ± 2.29	97.02 ± 2.33	1.442	0.154

Data presented as mean ± SD and numbers as appropriate

Independent sample *t* test; *p* value *>* 0.05 NS; *p value *<* 0.05 S

### PaCO_2_

There was no significant difference in postoperative PaCO_2_ between the two groups although PaCO_2_ was lower in the PCVVG group but fail to be statically significant (Table 6).

**Table 6:** Comparison between group A: VCV and group B: PCV-VG according to PaCO_2_

PaCO_**2**_	Group A: VCV (***n*** = 30)	Group B: PCV-VG (***n*** = 30)	***t*** test	***p*** value
Preoperative	33.57 ± 3.05	32.35 ± 4.31	1.266	0.210
After induction	34.24 ± 3.11	32.49 ± 4.40	1.779	0.080
Immediate postoperative	33.85 ± 3.72	33.39 ± 5.07	0.401	0.690
Postoperative at 30 min	34.05 ± 3.61	34.08 ± 4.77	0.027	0.978
Postoperative at 120 min	34.32 ± 3.68	34.85 ± 4.26	0.516	0.608
Postoperative at 240 min	35.07 ± 4.13	35.56 ± 4.60	0.434	0.665
Postoperative at 360 min	35.35 ± 3.48	35.51 ± 4.34	0.158	0.875

Data presented as mean ± SD

Independent sample *t* test; *p* value > 0.05 NS; *p value < 0.05 S

### Lung ultrasound

**Fig. 3:**
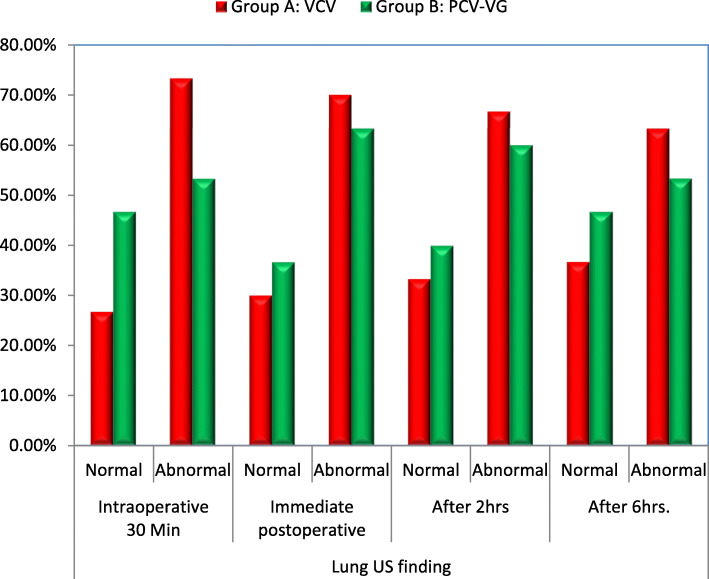
Bar chart between group A: VCV and group B: PCV-VG according to lung US finding

There was no significant difference in 30 min after induction, immediate, and 2 h and 6 h post-operative. Lung ultrasound findings between the two groups as shown in (Table 7). Although signs of atelectasis were low in group B all over the study, there was no significant difference between the two groups both showed atelectasis immediate, 2 h and 6 h postoperatively (Fig. 3).

**Table 7:** Comparison between group A: VCV and group B: PCV-VG according to lung US finding

Lung US finding	Group A: VCV (***n*** = 30)	Group B: PCV-VG (***n*** = 30)	***x***^**2**^	***p*** value
**Intraoperative 30 min**				
**Normal**	*8 (26.7%)*	*14 (46.7%)*	*2.540*	*0.111*
**Abnormal**	*22 (73.3%)*	*16 (53.3%)*		
*Absent A-lines*	*12 (40%)*	*10 (33.3%)*	*0.285*	*0.593*
*Presence B-lines*	*14 (46.7%)*	*11 (36.7%)*	*0.607*	*0.436*
*Lung pulse*	*17 (56.7%)*	*10 (33.3%)*	*3.263*	*0.0708*
**Immediate postoperative**				
**Normal**	9 (30.0%)	11 (36.7%)	0.298	0.585
**Abnormal**	21 (70.0%)	19 (63.3%)		
*Absent A-lines*	11 (36.7%)	9 (30.0%)	0.298	0.585
*Presence B-lines*	16 (53.3%)	14 (46.7%)	0.257	0.612
*Lung pulse*	16 (53.3%)	14 (46.7%)	0.257	0.612
**After 2 h**				
**Normal**	10 (33.3%)	12 (40%)	0.285	0.593
**Abnormal**	20 (66.7%)	18 (60%)		
*Absent A-lines*	7 (23.3%)	5 (16.7%)	0.402	0.526
*Presence B-lines*	14 (46.7%)	13 (43.3%)	0.069	0.793
*Lung pulse*	13 (43.3%)	12 (40%)	0.066	0.797
**After 6 h**				
**Normal**	11 (36.7%)	14 (46.7%)	0.607	0.436
**Abnormal**	19 (63.3%)	16 (53.3%)		
*Absent A-lines*	8 (26.7%)	6 (20.0%)	0.360	0.548
*Presence B-lines*	14 (46.7%)	13 (43.3%)	0.069	0.793
*Lung pulse*	14 (46.7%)	13 (43.3%)	0.069	0.793

Data presented as number and percentage, *p* value > 0.05 NS; *p value < 0.05 S

## Discussion

This study compared VCV with PCV-VG modes of ventilation. It shows that PCV-VG offered comparable results with VCV regarding the occurrence of postoperative atelectasis. In addition, there was an improvement in PaO_2_ when PCV-VG was used but failed to be statistically significant.

Atelectasis is an important determinant of postoperative pulmonary complications. Preventing atelectasis formation during the whole perioperative period will increase the O_2_ stores of the body and reduce the postoperative hypoxemia (Magnusson & Spahn, 2003).

Computed tomography (CT)-measured lung aeration has been the gold standard for the study of perioperative atelectasis. However, cumulative radiation exposure and the need to transport the patient to or from the radiology department limit its use even in the research setting (Brenner & Hall, 2007).

Lung ultrasonography plays a key role in the diagnosis of lung consolidation (atelectasis, pneumonia, etc.) as it enables not only the diagnosis at the patient’s bedside, but also the treatment response assessment in real time (Volpicelli et al., 2012). However, atelectasis is rarely detected early because of the lack of sensible and practical bedside methods for lung imaging. Lung ultrasound (LUS) has been proven to outperform chest radiography (CXR) in diagnosing common pulmonary pathologic abnormalities, such as pneumothorax, pleural effusion, and interstitial syndrome (Xirouchaki et al., 2011).

Yu et al. found that LUS showed a reliable performance in postoperative atelectasis, with a sensitivity of 87.7%, a specificity of 92.1%, and a diagnostic accuracy of 90.8%. In a study conducted on 46 patients without pulmonary comorbidities who were scheduled for elective neurosurgery, all included patients were within the American Society of Anesthesiologists’ (ASA) physical status classes I–II, and the surgical procedure was expected to take 2 h. The accuracy of LUS in detecting perioperative atelectasis was compared with thoracic CT as a gold standard. In patients in the supine position, the LUS provides a fast, reliable, and radiation-free method to identify perioperative atelectasis in adults (Yu et al., 2016).

Acosta and his colleagues investigated lung ultrasound for detecting postoperative lung atelectasis in 15 children aged 1 to 7 years old. After taking the reference lung MRI images, LUS was carried out; they found that anesthesia-induced atelectasis can be associated with LUS signs such as air bronchograms, absence of A-lines, presence of line B, absence of lung sliding, and presence of the pulse sign. Lung sonography showed a sensitivity of 88% (95% CI, 74 to 94%) and a specificity of 89% (95% CI, 83 to 94%) for the diagnosis of anesthesia-induced atelectasis (Acosta et al., 2014).

This study compared VCV with PCV-VG modes of ventilation. It shows that PCV-VG offered comparable results with VCV regarding the occurrence of postoperative atelectasis. In addition, there was an improvement in PaO_2_ when PCV-VG was used but failed to be statistically significant.

Our study was conducted on patients with no underlying lung pathology. We postulated that the effects of the different modes on the occurrence of lung atelectasis during different upper abdominal surgeries are better investigated in healthy patients. The complex alveolar pathology in restrictive and obstructive lung diseases and its interaction with the different modes of ventilation and different surgeries are absent; hence, the direct effects of the modes are better examined. All patients underwent a minimum of 5 cm H_2_O PEEP, which was continued throughout the surgery. PEEP was reported to prevent the development of postoperative atelectasis and hypoxemia, resulting in complications similar in the two study groups.

Regarding ABG, in our study, the PCV-VG mode showed improvement in oxygenation but was not of clinical significance over the VCV mode. Oxygenation was well maintained in the two groups of patients throughout the surgery. The PaO_2_ and SpO_2_ were comparable between the two groups. Although SpO_2_ was higher in PCV-VG group, this was clinically nonsignificant. The inspired oxygen concentration in our study was constant, and alveolar ventilation was uncompromised.

In agreement with our study, El-Rawas et al. found that regarding ABG, PCV-VG mode showed no advantage over VCV regarding intraoperative and postoperative PaO_2_ in a study conducted on sixty obese patients with normal preoperative pulmonary functions scheduled for upper abdominal laparotomies (El-Rawas et al., 2015).

Another agreement with our study, Lee et al., showed that arterial blood gas results did not differ significantly between the two groups receiving (PCV-VG and VCV) when comparing 36 patients undergoing lumbar spine surgery in the prone position (Lee et al., 2019).

Gad et al. investigated 80 female patients with body mass index (BMI) > 30 kg/m^2^ and with ASA classes I and II undergoing laparoscopic hysterectomy who were allocated randomly to either PCV-VG or VCV with equal ratio ventilation (ERV). The study showed that in agreement with our study, no significant differences were reported as regards the ABG analysis, oxygenation, and hemodynamic data between both groups. This could be explained by the issue stated that ERV improves oxygenation through increasing *P* mean only when alveoli are recruitable, and *P* mean which is considered a major determinant of arterial oxygenation did not record a significant difference between the studied groups (Gad et al., 2019).

Assad et al. supported our results regarding oxygenation and ventilation difference in a study conducted on 40 patients ASA physical status I and II patients that underwent elective laparoscopic surgery in Trendelenburg position reported that PCVVG ventilation mode showed no significant difference in oxygenation rates between the two modes. This finding may be explained by the similarity in *P* mean (Assad et al., 2016).

Our findings have been consistent with a study conducted on one-lung ventilation and respiratory failure patients. Cengiz et al. showed that PCV-VG had no effect on intra- and postoperative oxygenation values and does not reduce complications when comparing those two modes in 80 patients, aged 18–75 years, undergoing one-lung ventilation and lobectomies divided into two groups, as group 1 (PCV-VG) and group 2 (VCV) (Sahutoglu et al., 2018).

Song et al. also had similar results to our study. Their study was conducted on twenty-seven patients scheduled for thoracic surgery with one-lung ventilation (OLV) in the lateral decubitus position. The subjects received various modes of ventilation in random sequences during surgery, including VCV and PCV-VG. No difference in arterial oxygen tension was noted between the groups (*p = 0.063*). Patients in the last two studies were in similar criteria as our study patients with good respiratory function test results (Song et al., 2014).

Also, in agreement with our study, Guldager et al. conducted a study involved 44 patients suffering from acute respiratory failure, they compared PRVC (which is similar to PCVVG but present on the Siemens 300 ventilator) to VCV and showed that there was no statistically significant difference in PaO_2_ (Guldager et al., 1997).

On the other hand, Boules and Ghobrial evaluated the effects of PCV-VG and VCV during OLV. Forty patients undergoing elective thoracic surgery in the lateral position requiring at least 1 h of OLV. The mean PaO_2_ values increased significantly after lung inflation in the two groups comparably, but it was still significantly higher in the PCV-VG group (*p* value < 0.05) (Boules & Ghobrial, 2011). Other study was done by Pu et al. compared PCV-VG and VCV during OLV in thoracic surgery and showed a significant increase in PaO_2_ under PCV-VG (Pu et al., 2014).

The latter two studies used OLV; therefore, the improvement in PaO_2_ in the PCV-VG group in those studies may be due to the fact that using a lower airway pressure with a constant TV in a single lung may have a more profound effect than using it in double lung ventilation. Also, the fact that isolating a diseased lung may improve oxygenation could explain the presence of statistical significance in the previous studies and the absence of this difference in our study with healthy lungs.

A study was done by Toker et al. comparing 104 patients who underwent laparoscopic gynecologic surgery with a body mass index between 30 and 40 kg m^−2^ and was randomized to receive either VCV or PCV-VG ventilation. Mean PaO_2_ levels were significantly higher in the PCV-VG group than in the VCV group at every time point after pneumoperitoneum in the Trendelenburg position (Toker et al., 2019).

Regarding lung ultrasound in postoperative atelectasis, it has the advantage of allowing immediate diagnosis of complete atelectasis, before radiologic signs occur (Lichtenstein & Meziere, 2008).

In our study, we found no statistically significant difference between two groups regarding signs suggestive of atelectasis (absence of A-line and lung sliding, presence of B-lines and lung pulse) although signs were less in PCV-VG but failed to reach statically significant results.

Another study was done using a CT scan as a tool for detecting postoperative lung atelectasis, EL-RAWAS et al., in which 60 obese patients with normal preoperative pulmonary functions scheduled for upper abdominal laparotomies were randomized into two groups. Those in group A received VCV and those in group B received PCV-VG. Arterial blood gases (ABG) were obtained pre-, intra-, and postoperatively. Peak expiratory flow rate (PEFR) and CT chest were done pre- and postoperatively. There was no difference between the two groups as regards the occurrence of postoperative atelectasis. All preoperative CTs were free of atelectasis and the same number of patients developed postoperative basal lung atelectasis in both modes (El-Rawas et al., 2015).

In a study by Prella et al., comparing PCV (which uses decelerating flow like PCV-VG) to VCV in 10 patients with acute lung injury or ARDS, the CT chest showed that like in El Rawas et al. study, surface areas of the basal non-aerated zones were similar in the two modes; however, Prella et al. found that at the apex level, there was a significantly greater non-aerated area in VCV (Prella et al., 2002).

A different study was done by Kim et al. in a recent prospective randomized controlled trial. The PCV-VG group showed better LUS compared with the VCV group. They performed LUS at four different time points for each patient: before induction, 30 min after a semi-lateral position change, during supine repositioning before awakening, and 15 min after arrival to the post-anesthesia care unit (PACU), this may be due to different age groups showing senile emphysema compared to VCV, and PCV-VG seems to provide homogeneous ventilation and better lung aeration in the left and anterior compartments also different position lateral rather than supine position (in which the anatomical locations of the lung and heart may be influential) (Kim et al., 2019).

Our investigation presents some limitations, which need to be considered. Those limitations may have resulted in these negative findings; first, our study included patients with no lung pathology and has American Society of Anesthesiologist physical statuses I and II; therefore, our results apply only to healthy patients. In respiratory-compromised patients, the results of PCV-VG or VCV may show significant differences. Second, our study did not enroll any morbid obese patients which are a key factor in cardiorespiratory compromise developing in upper laparotomies. Third, our study was done on small numbers of patients which may be responsible for no clinical significant ratio. Moreover, we did not provide any guidance or solution when LUS was assessed to be positive for lung atelectasis and how to prevent it in the perioperative period.

## Conclusion

PCV-VG offered no significant advantage over VCV regarding the occurrence of the postoperative atelectasis. Further studies are required to determine the effect of each mode of ventilation on respiratory mechanics in different types of patients like obese patients, elder patients, large number, and patients with respiratory diseases. But, we prefer to use PCV-VG as postoperative hypoxia and atelectasis are much less in that mode. Further, large-scale studies are required to confirm these findings and to establish a definite conclusion.

## Data Availability

The datasets used and/or analyzed during the current study are available from the corresponding author on reasonable request.

## Abbreviations

ABG: Arterial blood gases
IV: Intravenous
LUS: Lung ultrasonography
MAC: Minimum alveolar concentration
OR: Operating room
PACU: Post-Anesthesia Care Unit
PaO_2_: Oxygen tension
PCV: Pressure-controlled ventilation
PCVG: Pressure-controlled volume guarantee
PCVVG: Pressure-controlled ventilation volume-guaranteed
PEEP: Positive-end expiratory pressure
PPC: Postoperative pulmonary complications
SD: Standard deviation
SPSS: Statistical package for social science
TE: Expiratory time
TI: Inspiratory time
TV: Tidal volume
VAS: Visual analog scale
